# Anisomycin has the potential to induce human ovarian cancer stem cell ferroptosis by influencing glutathione metabolism and autophagy signal transduction pathways

**DOI:** 10.7150/jca.83355

**Published:** 2023-05-05

**Authors:** Ying Xiong, Te Liu, Juan Chen

**Affiliations:** 1 Department of Obstetrics and Gynecology, Xinhua Hospital Affiliated to Shanghai Jiao Tong University School of Medicine, Shanghai 200092, China.; 2 Shanghai Geriatric Institute of Chinese Medicine, Shanghai University of Traditional Chinese Medicine, Shanghai 200031, China.; 3 Gongli Hospital Affiliated to the Second Military Medicical University in Pudong New Area of Shanghai City, Shanghai 200135, China.

**Keywords:** Ovarian cancer, Anisomycin, ferroptosis, glutathione, autophagy.

## Abstract

Ovarian cancer is a highly malignant gynecological tumor that seriously endangers women's health. Previously, we demonstrated that anisomycin significantly inhibited the activity of ovarian cancer stem cells (OCSCs)* in vitro* and *in vivo*. In this study, anisomycin treatment of OCSCs significantly reduced the content of adenosine triphosphate and total glutathione, increased the extent of lipid peroxidation, and increased malondialdehyde, and Fe^2+^ levels. The ferroptosis inhibitor Ferr-1 could significantly weaken the cytotoxicity of anisomycin. Subsequently, the cDNA microArray results suggested that anisomycin significantly reduced the transcription levels of gene clusters associated with protection from ferroptosis, such as those encoding members of the glutathione metabolism and autophagy signal transduction pathways. Bioinformatic analyses indicated that genes encoding core factors of these two pathways, and activating transcription factor 4 (*ATF4*), were significantly expressed in ovarian cancer tissues and correlated with poor prognosis. After overexpression or knockdown of *ATF4*, the ability of anisomycin to inhibit the proliferation and autophagy of OCSCs increased or decreased, respectively. Finally, analysis using a peripheral blood exosome database indicated that the contents of key factors (e.g., *ATF4*, *GPX4*, and *ATG3*) in peripheral blood exosomes from patients with ovarian cancer, were significantly higher than those of the healthy controls. Therefore, we hypothesized that anisomycin suppressed the expression of members of the glutathione metabolism and autophagy signal transduction pathways by downregulating the expression of ATF4. Moreover, anisomycin has the potential to induce human ovarian cancer stem cell ferroptosis. Overall, we confirmed that anisomycin has multiple targets and many mechanisms of action in inhibiting the activity of OCSCs.

## Introduction

Ovarian cancer is a highly malignant gynecological tumor that seriously endangers women's health 1, 2. In recent years, ovarian cancer has been observed in younger patients, and the degree of harm has also increased 1, 2. Studies have found that in ovarian cancer tissue samples, there are a group of cell subsets similar to embryonic stem cells, which express high levels of CD44, CD133, c-Kit (CD117), and other markers, displaying "stemness" similar to stem cells, but with the characteristics of tumor cells. They are characterized by high proliferation, high invasiveness, and high tumorigenicity, and thus are termed ovarian cancer stem cells (OCSCs) 3-5. OCSCs are highly heterogeneous and resistant to traditional chemotherapeutic drugs; therefore, it is particularly important to develop highly efficient, targeted drugs to kill OCSCs.

Anisomycin (3,4-pyrrolidinediol,2-[(4-methoxyphenyl)methyl]-,3-acetate, (2R,3S,4S) is an antibiotic purified from *Streptomyces lividans* 6. Anisomycin blocks peptide bond formation and inhibits protein synthesis by binding to the 60S ribosomal subunit 6-8. Anisomycin is a JUN N-terminal kinase (JNK) activator that enhances the phosphorylation of JNK 9, 10 and has significant cytotoxic effects on eukaryotes and some protozoa 6. Studies have pointed out that anisomycin not only promotes the production of amyloid β (Aβ42), but also inhibits the proliferation and invasion of various tumor cells 6, 7, 11-13. Yu *et al.* found that anisomycin inhibited Jurkat T cell proliferation by stimulating the p53, p21, and p27 signaling, and blocking signaling cells from entering the S phase and G2/M phase 14. Seo *et al.* reported that anisomycin could downregulate the expression of B-cell CLL/lymphoma 2 (Bcl2), CASP8 and FADD‑like apoptosis regulator (CFLIP), and myeloid cell leukemia sequence 1 (BCL2-related) (Mcl1), thereby enhancing renal tumor cell apoptosis 12. Meanwhile, Liu *et al.* demonstrated that anisomycin induces apoptosis in glucocorticoid-resistant acute lymphoblastic leukemia cells via the activation of phosphorylated mitogen-activated protein kinase p38 and JNK 10, 15. In addition, Li *et al.* revealed that anisomycin can induce glioma cell death *in vitro* by downregulating the catalytic subunit of inorganic pyrophosphatase 2 (PP2A) 15. Our previous study also illustrated that anisomycin inhibits the proliferation and invasion of human OCSCs (HuOCSCs) by stimulating the expression of the long noncoding RNA (lncRNA) *BACE1-AS1* and upregulating the concentration of intracellular Aβ42 7. Additionally, we demonstrated that anisomycin inhibited the downstream activation of the Notch1 pathway by attenuating the molecular sponge effect of the lncRNA-*Meg3*/miR-421/platelet derived growth factor receptor alpha (PDGFRA) axis, ultimately inhibiting the angiogenesis, proliferation, and invasion of ovarian cancer cells 13. Thus, anisomycin is a promising chemotherapeutic drug.

Ferroptosis is a newly recognized iron-dependent, programmed cell death mechanism that is different from apoptosis, necrosis, and autophagy 16-21. The primary mechanism of ferroptosis is that under the action of ferrous iron or ester oxygenase, unsaturated fatty acids highly expressed on the cell membrane are induced to undergo liposome peroxidation, thereby inducing cell death. Ferroptosis is also manifested in the antioxidant system, and it appears that the expression of glutathione peroxidase 4 (GPX4) decreases in the antioxidant system (lipid peroxide) 16-21. Studies have found that when iron ions are continuously input into cells and are maintained in the form of divalent iron, an iron overload state is reached in which ubiquitin can initiate liposome peroxidation. Lipoperoxidized liposomes can significantly decrease the activity of GPX4* in vivo* and induce ferroptosis 17, 20. In addition, studies have found that ferroptosis suppressor protein 1 (Fsp1) is a glutathione-independent ferroptosis inhibitor, and its Fsp1/CoQ10/NAD(P)H signaling pathway acts as an independent parallel system, which, together with GPX4 and glutathione, inhibits phospholipid peroxidation and ferroptosis 22, 23. When cells undergo ferroptosis, cell mitochondria become smaller, membrane density increases, and the number of cristae decreases, accompanied by increased cytoplasmic lipid peroxidation and increased levels of reactive oxygen species (ROS) 16, 18, 21. In addition, cystine-glutamate antiporter (system Xc-)/GPX4 is important in ferroptosis: pharmacological inactivation of system Xc- or GPX4 can induce ferroptosis, suggesting the crucial roles of the glutathione-dependent antioxidant defenses in preventing ferroptosis 16, 17, 21, 22. High concentrations of extracellular glutamate, erastin, or other system Xc- inhibitors block intracellular cystine/cysteine uptake to induce ferroptosis 16, 17, 21, 22. It is important to note that system Xc- function is regulated by glutamate levels, because glutamate is exchanged for cystine in a 1:1 ratio by system Xc-. Accordingly, high extracellular concentrations of glutamate block system Xc- activity, inhibit cystine uptake, and drive ferroptosis 16, 21. Increasing numbers of studies have highlighted ferroptosis as a potential new means of tumor therapy.

Based the above evidence, in the present study, we aimed to use gene expression profiling chips to screen for gene expression closely related to ferroptosis. In addition, combined with a large sample database of patients with tumors and bioinformatic analysis methods, we aimed to explore whether anisomycin has the potential to induce OCSC ferroptosis by inhibiting glutathione metabolism and autophagy signaling pathways. Through this study, we attempted to confirm that anisomycin has multiple targets and many mechanisms of action in inhibiting the activity of OCSCs. Meanwhile, our results offer potential diagnostic markers for early screening of ovarian cancer.

## Material and methods

### Isolation and culture of primary HuOCSCs

The experiment was performed according to the previously described methods 4, 13. Surgically isolated tissues from four patients with ovarian cancer were minced, digested with 0.25% trypsin (Gibco, Gaithersburg, MD, USA), and centrifuged at 1500 r/min for 5 minutes. The cell precipitates were collected and incubated with mouse anti‑human CD133-fluorescein isothiocyanate (FITC) antibodies and rabbit anti-human CD44-allophycocyanin (APC) antibodies (e-Bioscience Inc., San Diego, CA, USA) *in vitro* at 4 °C for 30 minutes. Next, CD44+/CD133+ OCSCs were sorted from the samples using flow cytometry (BD FACSAria; BD Biosciences, San Jose, CA, USA). All HuOCSC cells were cultured under the same conditions until passage 2 before being subjected to the indicated treatments.

### HuOCSC treatment

According to previous research 4, 13, anisomycin (Sigma-Aldrich, St. Louis, MO, USA) was applied at a final concentration of 31.8 µM (IC_50_ value) in all assays. The same volume of dimethyl sulfoxide (DMSO; Sigma-Aldrich) was applied to the cells as the control (Ctrl) group. The concentrations of Ferrostatin-1(Ferr-1, 4.0 μM) and Erastin (Erast, 1.0 μM) were the same as those cited in previous studies 19, 20.

### 3-(4,5-Dimethylthiazol-2-yl)-2,5-diphenyltetrazolium-bromide (MTT) assay

According to previous research 13, 10 μL of MTT solution (Sigma-Aldrich) was added to each group of cells and incubated at 37 °C for 3 h. The formula to calculate the cell proliferation inhibition rate (%) was (1 - the OD value of the sample group / the OD value of the control group) × 100%.

### The malondialdehyde (MDA) assay

The relative MDA concentration in cell or tumor lysates was assessed using a malondialdehyde Assay Kit (Abcam, Cambridge, MA, USA; #ab118970) according to the manufacturer's instructions and previous studies 19, 21. MDA in the sample reacts with thiobarbituric acid (TBA) to generate an MDA-TBA adduct. The MDA-TBA adduct can be quantified colorimetrically (OD at 532 nm). C11-BODIPY dye (Thermo Fisher Scientific, Waltham, MA, USA) was used to detect lipid peroxidation in cells. Oxidation of the polyunsaturated butadienyl portion of the dye results in a shift of the fluorescence emission peak from approximately 590 to approximately 510 nm.

### Adenosine triphosphate (ATP) assay

The ATP assay was performed according to the manufacturer's protocol from the Enhanced ATP Assay Kit (Beyotime, Shanghai, China) and in accordance to previous studies 19, 21. Two hundred microliters of the sample lysate were added to 1 × 10^6^ cells/mL and thoroughly mixed by pipetting. The mixture was then centrifuged at 12,000 × *g* for 5 minutes at 4 °C and the supernatant was collected. At the same time, the ATP standard solutions were set up and adjusted to the following concentrations: 0.01, 0.03, 0.1, 0.3, 1, 3, and 10 μM, respectively, and then tested simultaneously with the samples. Fresh testing solutions were prepared as required by the kit's protocol. The ATP testing solution (100 μL) was added to each of the testing wells and standard wells and incubated at room temperature for 5 minutes. Then, 20 μL of the test sample or standard solution was added to the wells and quickly mixed. After 5 seconds at room temperature, the relative light unit (RLU) values were measured using a luminometer (Model 680, Bio-Rad, Hercules, CA, USA).

### cDNA microarray analysis

Total RNAs from each group of cells were labeled using Agilent's Low RNA Input Fluorescent Linear Amplification Kit (Agilent, Santa Clara, CA, USA). Cy3-dCTP or Cy5-dCTP was incorporated during reverse transcription of 5 μg of total RNAs into cDNA. Different fluorescently labeled cDNA probes were mixed with 30 μL of hybridization buffer (3 × SSC, 0.2% SDS, 5 × Denhardt's solution, and 25% formamide) and applied to the microarray (CapitalBio human mRNA microarray V2.0, CapitalBio, Beijing, China) followed by incubation at 42 °C for 16 hours. Following hybridization, the slide was washed with 0.2% SDS/2 × SSC at 42 °C for 5 minutes and then washed with 0.2 × SSC at room temperature for 5 minutes. The fluorescent images of the hybridized microarray were scanned using an Agilent Whole Human Genome 4×44 microarray scanner system. Images and quantitative data of the gene-expression levels were analyzed using Agilent's Feature Extraction (FE) software, version 9.5 13.

### Bioinformatic prediction and analysis

A total of 426 patients with ovarian cancer (T) and 88 non-ovarian cancer patients (N) from the Gene Expression Profiling Interactive Analysis (GEPIA) (http://gepia.cancer-pku.cn/index.html) database were included as the study patient cohorts. These data on patient cohorts were then used for gene-expression profile analysis, pathological stage-plot analysis, multiple gene-comparison analysis, and gene-correlation analysis using the GEPIA online tool (Affymetric id/Gene symbol: GPX4, glutathione Synthetase (GSS), Kelch like ECH associated protein 1 (KEAP1), sequestome 1 (SQSTM1), solute carrier family 7 member 11 (SLC7A11), autophagy related 5 (ATG3), ATG5, and ATG4D) 24. The Kaplan-Meier plotter online tool (https://kmplot.com/analysis/) was used to analyze and plot survival curves from patient cohorts for a total of 1,435 patients with ovarian cancer 25.

The list of differentially expressed genes then underwent Gene Ontology (GO) and pathway enrichment analyses using the Protein ANalysis THrough Evolutionary Relationships (PANTHER) classification system (http://www.pantherdb.org/about.jsp) 26,27. The STRING database (https://cn.string-db.org) was used to construct the protein-protein interaction (PPI) network 28.

The online software ALGGEN PROMO (http://alggen.lsi.upc.es/cgi-bin/promo_v3/promo/promoinit.cgi) and Tomtom (https://meme-suite.org/meme/tools/tomtom) were used to predict basic sequence motifs in gene promoters and transcription factor binding sites 29-31.

The online tool exoRBase 2.0 (http://www.exorbase.org/) was used to identify ovarian cancer-associated protein levels in peripheral blood exosomes and to predict diagnostic markers 32. Patients with ovarian cancer (n = 30) and healthy controls (n = 118) from the exoRBase 2.0 database were included in the study patient cohorts.

### Iron assay

The measurements of the ferrous iron (Fe^2+^) were performed using an Iron Analysis Kit (AB83366, Abcam, Cambridge, UK) according to the manufacturer's instructions. First, cells were homogenized with five volumes of Iron Assay buffer. The insoluble material was removed via centrifugation at 13000 × *g* at 4°C to obtain the supernatant for the assay. To estimate Fe^2+^ iron, 50 μL of supernatant was incubated with 50 μL of Iron Reducer Assay buffer in a 96-well microplate for 30 minutes at room temperature. Second, 50 μL of assay buffer was incubated with 200 μL of reagent mix in the dark for 30 minutes at room temperature. Subsequently, 100 μL of Iron Probe was added into the standard and test samples and thoroughly mixed, followed by incubation for 1 h at room temperature in the dark. At the end of the assay, the absorbance was determined using a microplate reader (Model 680, Bio-Rad) 33-35.

### Extraction of total RNA and quantitative real-time reverse transcription polymerase chain reaction (qRT-PCR)

Total RNA from each group of cells was extracted using the Trizol reagent according to the manufacturer's instructions. Total RNA was treated with DNase I (Sigma-Aldrich), quantified, and reverse transcribed into cDNA using the ReverTra Ace-α First Strand cDNA Synthesis Kit (TOYOBO, Osaka, Japan). Quantitative real-time PCR (qPCR) was performed using the cDNA as the template with a RealPlex4 real-time PCR detection system from Eppendorf Co. Ltd. (Hamburg, Germany). SYBR Green Real‑Time PCR Master Mix (TOYOBO) was used to provide the fluorescent dye in the nucleic acid amplification. qPCR was completed with 40 amplification cycles as follows: denaturation at 95 °C for 15s, annealing at 58 °C for 30s, and extension at 72 °C for 42s. The relative gene expression levels were calculated using the 2^-ΔΔCt^ method (Ct, cycle threshold) (ΔCt = Ct_genes-Ct_18sRNA; ΔΔCt = ΔCt_all_groups-ΔCt_control_group). The mRNA expression levels were normalised to the expression level of 18s rRNA. The primers were as follows: BECN1-FP:5'-ACCTCAGCCGAAGACTGAAG-3'; BECN1-RP:5'-AACAGCGTTTGTAGTTCTGACA-3'; ATG5-FP:5'-AAAGATGTGCTTCGAGATGTGT-3'; ATG5-RP:5'-CACTTTGTCAGTTACCAACGTCA-3'; ATG12-FP:5'-TAGAGCGAACACGAACCATCC-3'; ATG12-RP:5'-CACTGCCAAAACACTCATAGAGA-3'; ATG3-FP:5'-GATGGCGGATGGGTAGATACA-3'; ATG3-RP:5'-TCTTCACATAGTGCTGAGCAATC-3'; ATG4D-FP:5'-TATGGGCCATCGCTAGTGG-3'; ATG4D-RP:5'-CATACACGGGGTTGAGAGTCT-3'; SQSTM1-FP:5'-AAGCCGGGTGGGAATGTTG-3'; SQSTM1-RP:5'-CCTGAACAGTTATCCGACTCCAT-3'.

### Cell staining and flow cytometry

The experiment was performed according to the instruction manual of the Cell Death Detection Kit (Beyotime Biotechnology, Jiangsu, China). Briefly, the cells were washed with phosphate-buffered saline (PBS) once, centrifuged to remove residual medium, and gently resuspended in 195 μL of binding solution. Next, 5 μL of Annexin V-FITC, or Caspase 1-FITC was added, and the sample was gently mixed. Finally, 10 μL of propidium iodide (PI) staining solution was added, and the sample was gently mixed and incubated at 20 °C in the dark for 30min. The cells were then detected using a flow cytometer (Cytomics FC 500, BECKMAN, Brea, CA, USA).

### Statistical analysis

Each experiment was performed at least three times, and the data are shown as the mean ± SD where applicable. Differences between two groups were evaluated using Student's *t*-test. The probability of *p* < 0.05 was considered statistically significant. For the analysis of variance (ANOVA) and linear models for microarray data (LIMMA) options, genes with |Log2FC| Cutoff > 1 and q < 0.01 compared with pre-set thresholds were considered differentially expressed genes. In the survival analysis, log-rank tests were performed and a log-rank *p* < 0.01 was considered statistically significant. GraphPad Prism 7 software was used for the statistical calculations (GraphPad Inc., La Jolla, CA, USA).

## Results

### Anisomycin can induce ferroptosis in HuOCSCs

**Figure 1:**
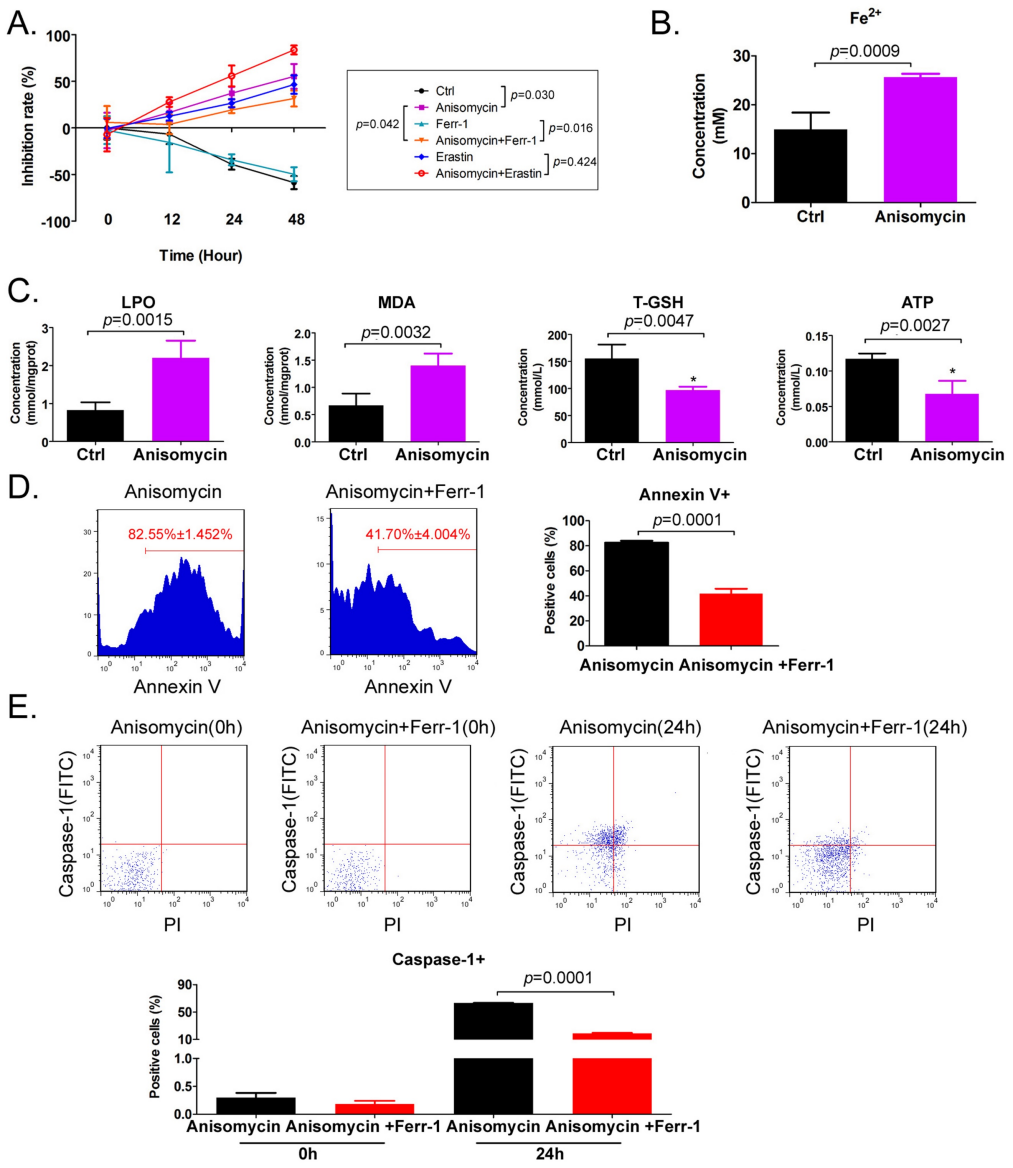
** Anisomycin has the potential to induce ferroptosis in HuOCSCs. (A)** MTT results suggesting that anisomycin inhibits the proliferation of HuOCSCs *in vitro*. **(B)** Anisomycin induces high Fe^2+^ levels in HuOCSCs.** (C)** Effects of anisomycin on lipid peroxides and ATP production in HuOCSCs. **(D)** The Annexin V+ HuOCSCs (apoptotic cells) were determined by immunofluorescence staining combined with flow cytometry. **(E)** The Caspase-1+ HuOCSCs (necroptotic cells) were determined by immunofluorescence staining combined with flow cytometry assay.

The results of the MTT assay indicated that treatment of HuOCSCs with the IC50 concentration of anisomycin significantly inhibited cell proliferation, and the extent of this inhibition correlated positively with the treatment time (Figure 1A). Subsequently, following treatment of the HuOCSCs with Ferr-1 combined with anisomycin, the growth inhibition rate of the cells over time was significantly lower than that of the anisomycin alone group (Figure 1A). Meanwhile, the results of the adherent cell count were consistent with those of the MTT assay (Supplementary data Figure S1). Following treatment of HuOCSCs with the ferroptosis agonist erastin combined with anisomycin, the growth inhibition rate over time was significantly higher than that of the cells in the anisomycin alone and erastin alone groups (Figure 1A). The intracellular Fe^2+^ concentration in the anisomycin-treated HuOCSC group was significantly higher than that in the control group (Figure 1B). Moreover, the contents of ATP and total glutathione (T-GSH) in the anisomycin-treated HuOCSCs were significantly decreased, while the contents of lipid peroxidation (LPO) and MDA were significantly higher than those in the control cells (Figure 1C). In addition, the results of cell immunofluorescence staining combined with flow cytometry indicated that the percentage of annexin V+ HuOCSCs (apoptotic cells) of the anisomycin+Ferr-1 treated group was significantly lower than that in the anisomycin only treated group (Figure 1D). The results revealed that the percentage of Caspase-1+ HuOCSCs (necroptotic cells) of the anisomycin+Ferr-1 treated group was significantly lower than that in the anisomycin only treated group (Figure 1E). Thus, the results suggest that the use of Ferr-1 partially reversed the inhibitory effect of anisomycin on the viability of HuOCSCs cells, the decreased the extent of apoptosis, and necroptosis. As such, the results suggest that the use of Ferr-1 partially reverses the apoptotic and necroptotic effects of anisomycin on HuOCSCs. Moreover, anisomycin could significantly induce the accumulation of lipid peroxides and ferrous ions in HuOCSCs, and inhibit the functions of cellular detoxification and energy production. Therefore, anisomycin has the potential to induce cellular ferroptosis.

### Anisomycin significantly inhibits the expression of ferroptosis-related genes in HuOCSCs

**Figure 2:**
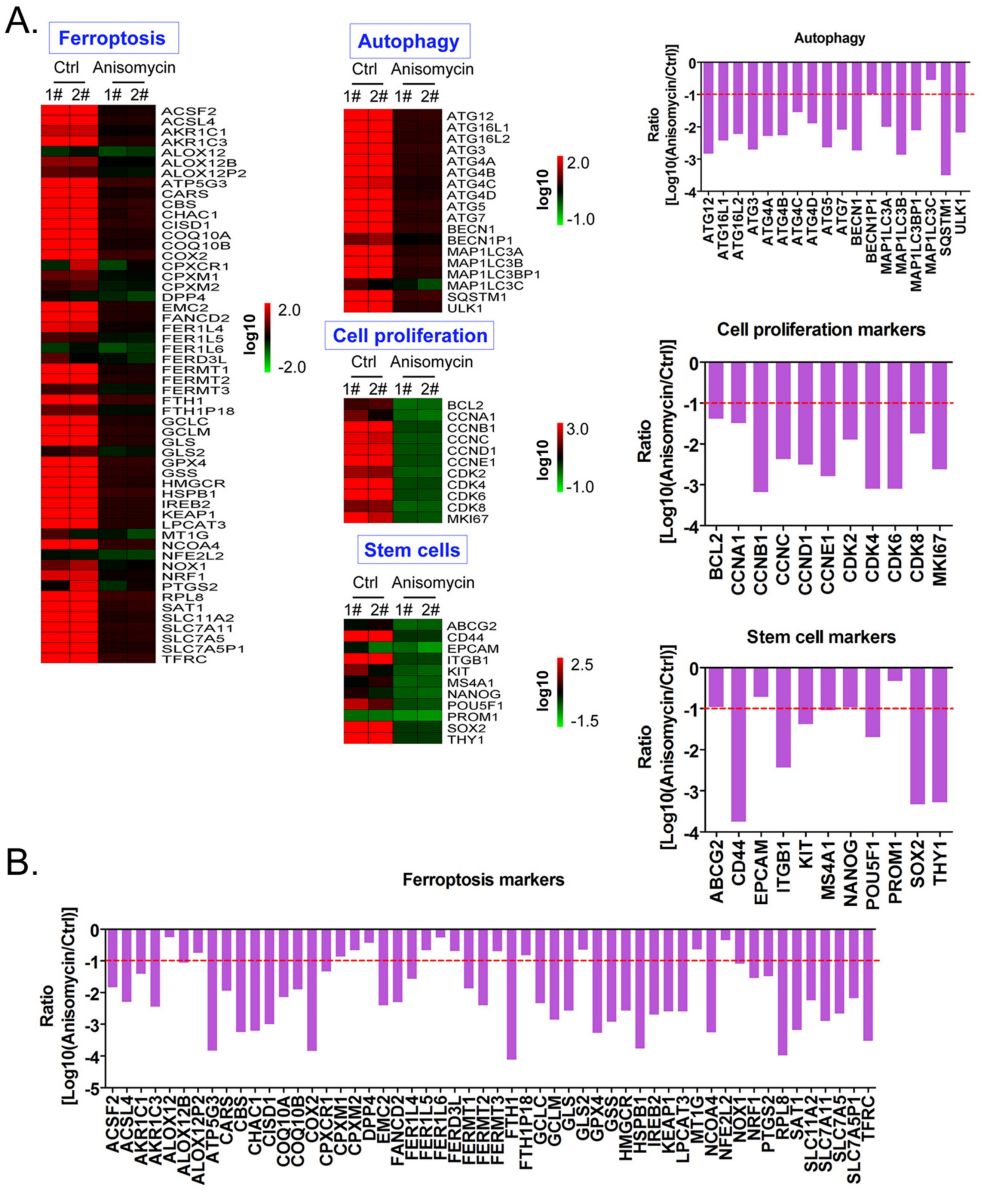
** Anisomycin significantly inhibits the expression of ferroptosis-related genes in HuOCSCs. (A)** Heatmap statistical analysis of ferroptosis-related gene transcript levels in the cDNA microarray data. **(B)** Anisomycin significantly inhibited the expression of ferroptosis-related genes in HuOCSCs.

**Figure 3:**
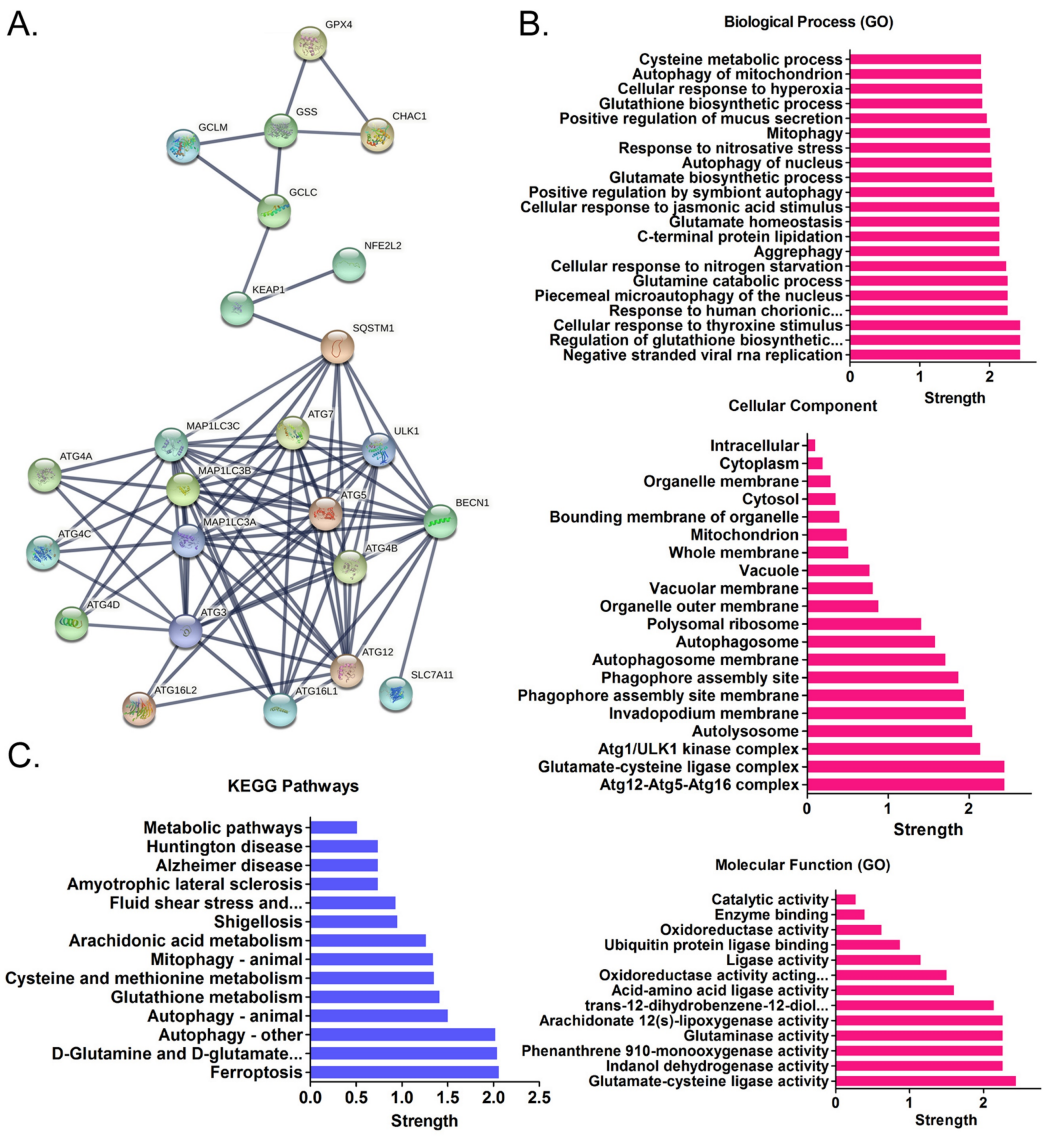
** Functional prediction of differentially expressed genes. (A)**Results of PPI network prediction. **(B)** Results of GO analysis prediction.** (C)** Results of pathway analysis prediction.

Using cDNA microarray high-throughput detection technology, we analyzed the differences in gene expression levels in each group of cells. A total of 28,455 genes were detected, and the expression levels of 94 genes closely related to ferroptosis were primarily analyzed. These genes were derived from categories such as ferroptosis, cell proliferation, autophagy, and stem cell biomarkers (Figure 2A). The results indicated that the expression levels of the above 94 genes in the anisomycin-treated HuOCSCs were significantly lower than those in the control cells (Log10(anisomycin/control) < -1.0, Figure 2B). Thus, the results suggested that anisomycin significantly inhibits the expression of ferroptosis-related genes in HuOCSCs. The results of the PPI network prediction showed that the proteins encoded by the 94 ferroptosis-related genes can form two relatively independent PPI networks, which are related to glutathione metabolism and the autophagy signal transduction pathway, respectively. The networks are interconnected via the Kelch-like ECH associated protein 1 (KEAP1) (Figure 3A). The PPI prediction results indicated that glutathione metabolism and autophagy are inseparable in terms of physiological activity. Subsequently, GO analysis showed that the 94 ferroptosis-related genes have different biological effects, such as the regulation of the glutathione biosynthetic process and cellular response to thyroxine stimulus (biological process); glutaminase activity and arachidonate 12(s)-lipoxygenase activity (molecular function); and the ATG12-ATG5-ATG16 complex and the glutamate-cysteine ligase complex (cellular component) (Figure 3B). Pathway analysis indicated that 47 of the 94 genes are involved in metabolic pathways such as ferroptosis, D-glutamine and D-glutamate metabolism, and autophagy (Figure 3C).

### Expression levels of glutathione metabolism and autophagy signal transduction pathway members are closely related to the development and prognosis of ovarian cancer

**Figure 4:**
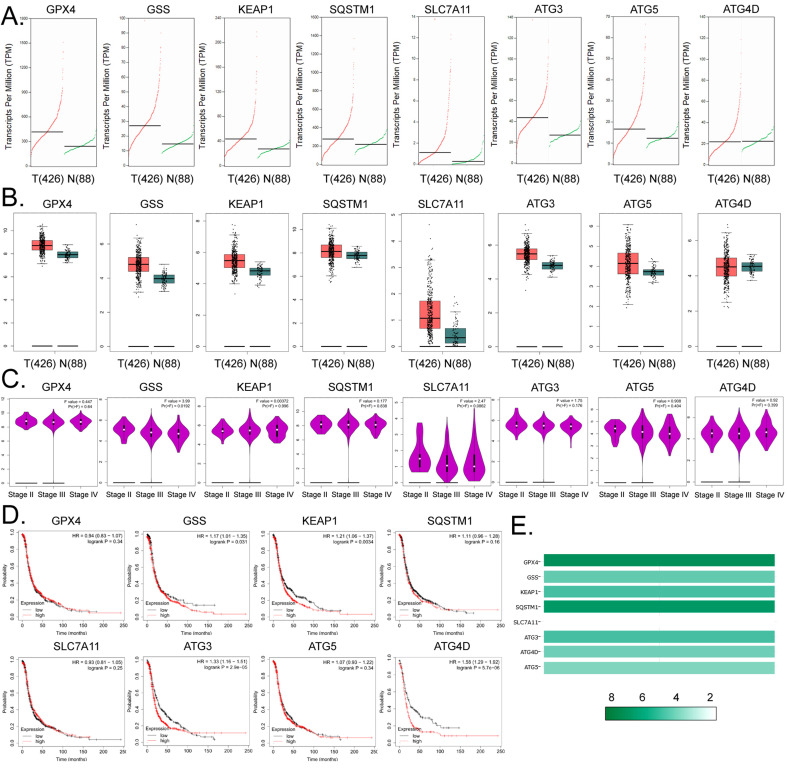
** Expression levels of members of the glutathione metabolism and autophagy signal transduction pathways are closely related to the development and prognosis of ovarian cancer. (A)**The results of transcript-level prediction analysis. **(B)** The results of the predictive analysis of gene expression levels. **(C)** The relationship between the expression levels of core factors of the glutathione metabolism, and autophagy signal transduction pathways, and the grade of ovarian cancer in patients. **(D)** The results of Kaplan-Meier Plotter analysis. **(E)** Differences in the expression levels of the core factors of the glutathione metabolism and autophagy signal transduction pathways between ovarian tissues and healthy tissues.

Based on the above findings, we further explored the link between factors related to glutathione metabolism and autophagy signal transduction pathways and the development and prognosis of ovarian cancer. The results of cDNA expression profile analysis indicated that six genes, including *GPX4*, *GSS*, *KEAP1*,* SQSTM1*, *SLC7A11*, *ATG3*, and *ATG5*, were highly expressed in tumor tissues from patients with ovarian cancer (Figure 4A, 4B). The statistical analysis of the pathological stage plot demonstrated that the expression level of *GSS* correlated negatively with the stage of ovarian cancer; however, he expression levels of the other factors showed no significant difference between the stages of ovarian cancer in patients (Figure 4C). Kaplan-Meier plot analysis indicated that the expression levels of *GSS*, *KEAP1*, *ATG3*, and *ATG4D* correlated significantly and negatively with the survival of patients with ovarian cancer (Figures 4D). In addition, in ovarian cancer tissue samples, the expression levels of *GPX4* and *SQSTM1* were about 8-fold higher than those of the other genes (Figure 4E). These results suggest that the expression levels of core factors of the autophagy signal transduction pathway are closely related to ferroptosis and have a certain correlation with the development and prognosis of ovarian cancer.

### ATF4 regulates the expression of glutathione metabolism and autophagy signal transduction pathway members and affects the malignancy of ovarian cancer

**Figure 5:**
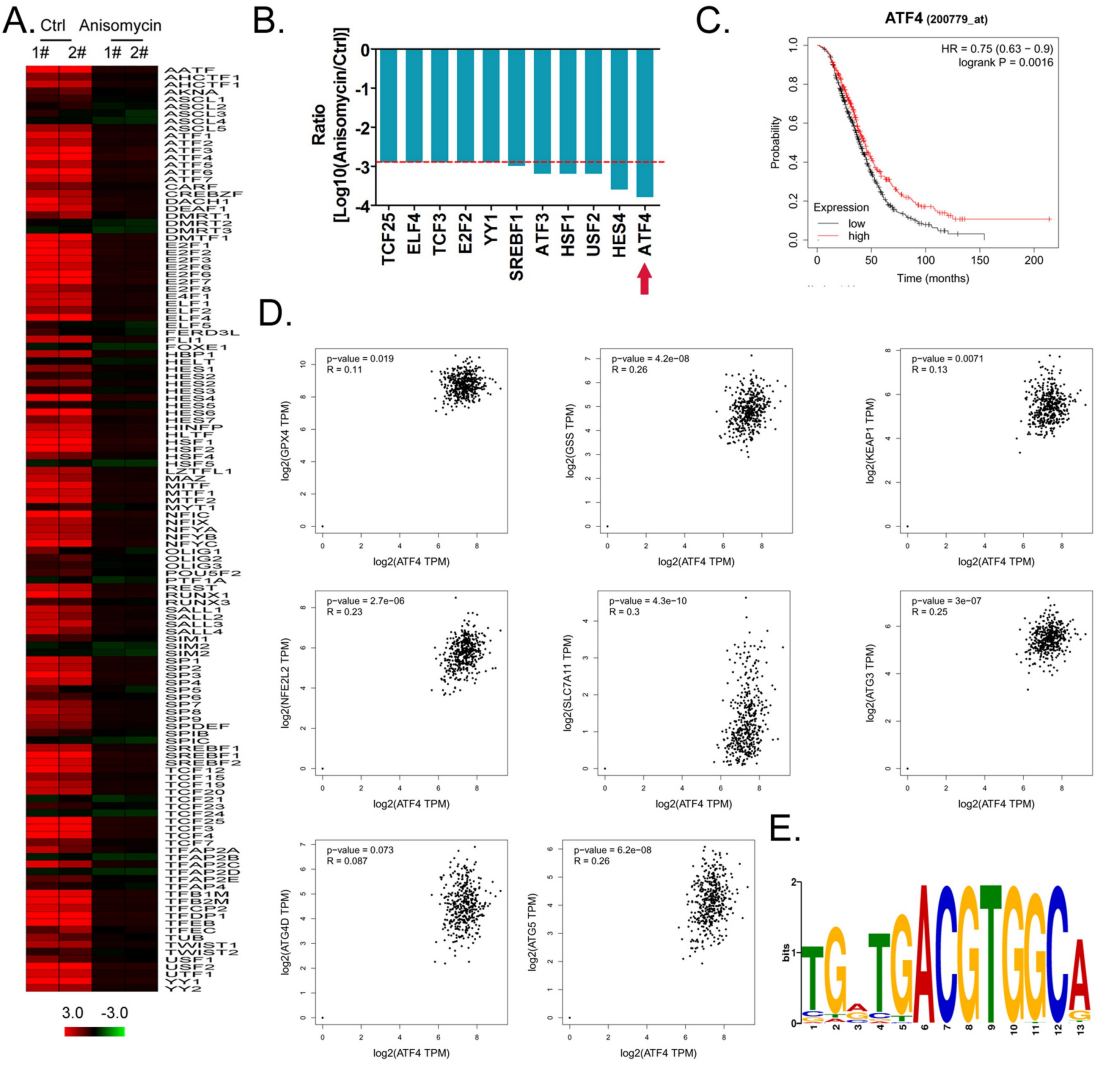
** ATF4 is a key transcription factor that regulates the expression of members of the glutathione metabolism and autophagy signal transduction pathways and affects the malignancy of ovarian cancer. (A)** Heatmap statistical analysis of transcription factor expression levels from the cDNA microarray data. **(B)** Anisomycin significantly inhibits the expression of ATF4. **(C)** The results of Kaplan-Meier Plotter analysis. **(D)** The relationship between the expression level of *ATF4* and the grade of ovarian cancer in patients. **(E)** The results of transcription factor motif analysis.

Considering the central dogma, that the first step of gene expression is DNA transcription and the activation of transcription requires transcription factors, we analyzed the results related to the expression levels of 128 transcription factors in the cDNA microarray data in detail. We found that the expression levels of 11 transcription factor genes (*TCF25*, *ELF4*, *TCF3*, *E2F2*, *YY1*, *SREBF*1, *ATF3*, *HSF1*, *USF2*, *HES4*, and *ATF4*) in the anisomycin-treated HuOCSC group were significantly lower than those in the control samples (Log10[anisomycin/control] <-2.9, Figure 5A, 5B). The Kaplan-Meier plot analysis showed that *ATF4* also had a significant negative correlation with the survival of patients with ovarian cancer (Figure 5C). The results of multiple gene correlation analyses indicated that there was a significant linear relationship (positive correlation) between the expression of *ATF4* in ovarian cancer tissue and the expression levels of *GPX4*, *GSS*, *KEAP1*, *NFE2L2*, *SLC7A11*, *ATG3*,* ATG4D*, and *ATG5* (Figure 5D). Finally, using transcription factor motif analysis, we observed that there is more than one binding site motif for ATF4 in the upstream promoter (approximately 1000 bp upstream of the transcription start site) regions of *GPX4*, *GSS*, *NFE2L2*, *ATG3*, *ATG4D*, and other genes, and all the sites have the [TGACGTGGC(A/G)] basic motif sequence (Figure 5E).

**Figure 6:**
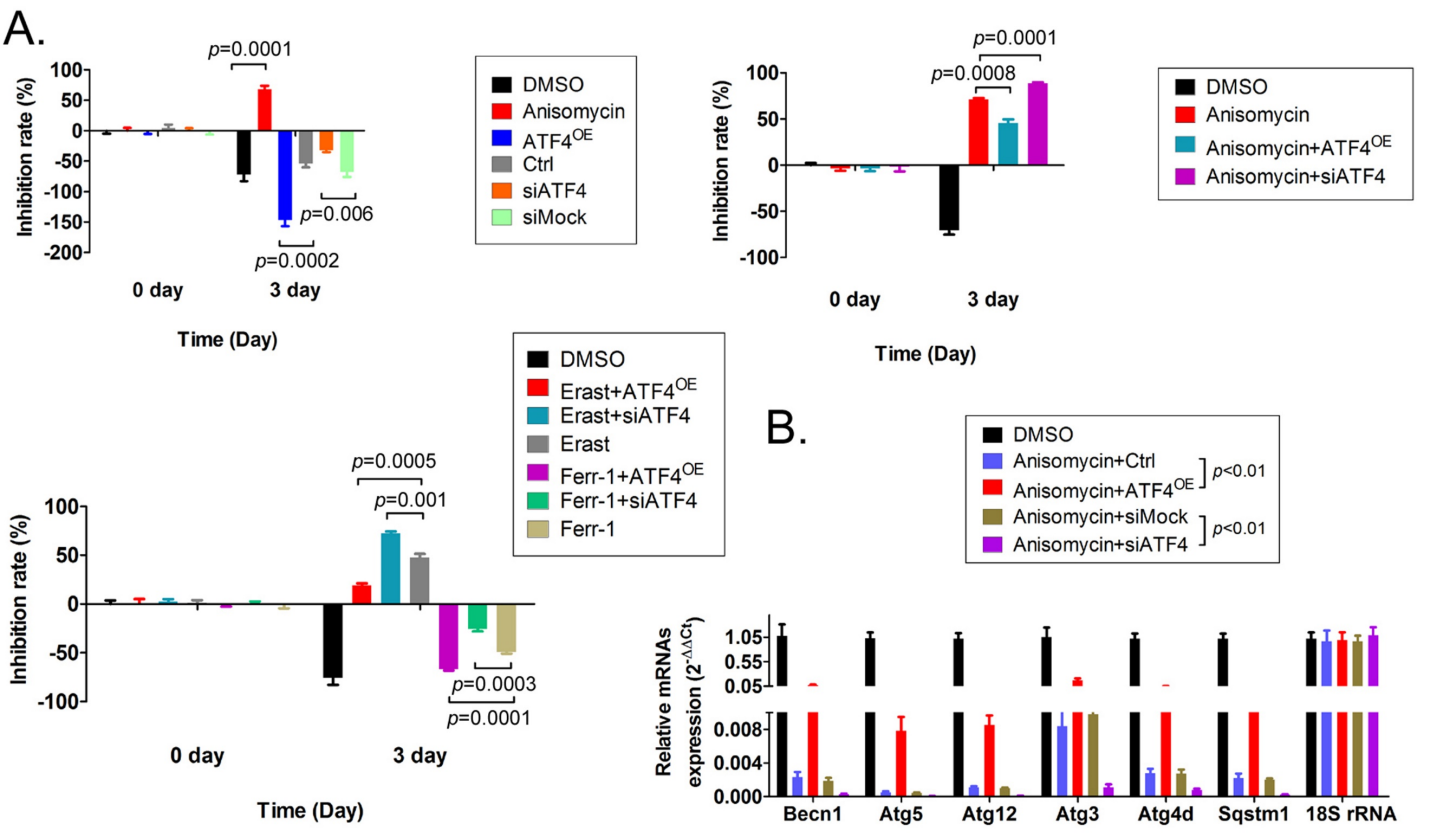
** Effects of overexpression or knockdown for *ATF4* on ferroptosis and autophagy pathways in HuOCSCs. (A)** The results of the MTT assay. **(B)** The results of the qPCR assay.

To verify the relationship between the expression level of ATF4 and autophagy and ferroptosis, additional MTT and qRT-PCR assays were carried out. The results of the MTT assay showed that overexpression of *ATF4* significantly promoted the proliferation of HuOCSCs *in vitro* and reduced the cell proliferation inhibition rate (Figure 6A). However, silencing the expression of endogenous *ATF4* using a small interfering RNA (siATF4) significantly increased the cell proliferation inhibition rate (Figure 6A). Moreover, the cell proliferation inhibition rate of HuOCSCs overexpressing ATF4 and treated with anisomycin was significantly lower than in HuOCSCs treated only with anisomycin (Figure 6A). Silencing the expression of the endogenous *ATF4* showed the opposite results (Figure 5A). In addition, *ATF4* overexpression reduced the inhibition of HuOCSC proliferation caused by the ferroptosis agonist erastin (Figure 5A), whereas siATF4 has the opposite effect (Figure 6A). Therefore, the above results suggested that ATF4 could suppress HuOCSC ferroptosis. In contrast, the results of qRT-PCR revealed that the expression levels of key genes (*BECN1*, *ATG3*, *ATG4D*, *ATG5*, *ATG12*, and *SQSTM1*) of the autophagy signal transduction pathway in HuOCSCs overexpressing *ATF4* and treated with anisomycin were significantly higher than those in the control group (Figure 6B). By contrast, the expression levels of these key autophagy genes in siATF4 transfected HuOCSCs treated with anisomycin were significantly lower than those in the control group (Figure 6B). Thus, the qRT-PCR results suggested that ATF4 promotes the autophagy of HuOCSCs. Meanwhile, ATF4 is linked two relatively independent events of autophagy with ferroptosis in HuOCSCs.

### Contents of ATF4 and key factors of glutathione metabolism and autophagy signal transduction pathways in peripheral blood exosomes from patients with ovarian cancer are significantly higher than those of healthy controls

**Figure 7:**
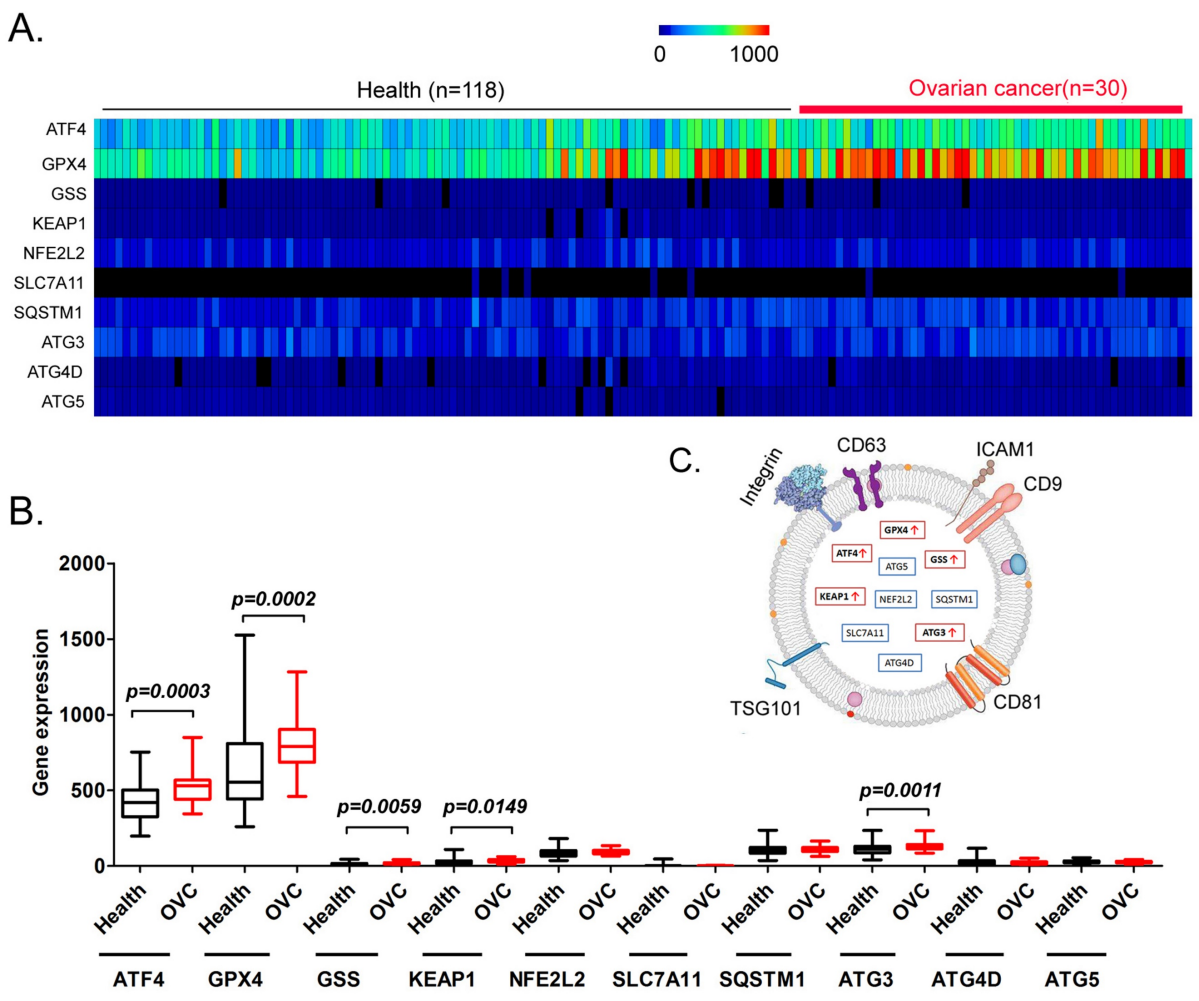
** Analysis of exosomes in the peripheral blood of patients with ovarian cancer. (A)** Heatmap analysis of ATF4 and key factors of glutathione metabolism and autophagy signal transduction pathways in peripheral blood exosomes from patients with ovarian cancer. **(B)** The levels of ATF4 and key factors of glutathione metabolism and autophagy signal transduction pathways in the peripheral blood exosomes of patients with ovarian cancer were significantly higher than those of the healthy controls. **(C)** Peripheral blood exosomes from patients with ovarian cancer.

Finally, we explored whether factors such as ATF4, GPX4, GSS, KEAP1, NFE2 like BZIP transcription factor 2 (NEF2L2), SLC7A11, SQSTM1, ATG3, ATG4D, and ATG5 have potential as non-invasive diagnostic markers for ovarian cancer. We analyzed the differences in the content of the above factors in the peripheral blood exosomes of patients with ovarian cancer and healthy controls (Figure 7A). The results of the data analysis indicated that the contents of ATF4, GPX4, GSS, KEAP1, and ATG3 in the peripheral blood exosomes of patients with ovarian cancer were significantly higher than those of the healthy controls (Figure 7B, 7C). The results suggested that the above factors have potential as non-invasive diagnostic markers to predict the occurrence and development of ovarian cancer.

## Discussion

Anisomycin has been proven to have a cytotoxic effect on a variety of tumors 6, 7, 13, 14. In addition, we previously determined that anisomycin can significantly inhibit the proliferation, invasion, and angiogenesis of HuOCSCs *in vitro* and *in vivo* 7, 13. It has also been revealed that biologically, anisomycin regulates multiple targets such as JNK and lncRNAs 7, 9, 10, 13. These studies showed that the cytotoxicity of anisomycin comes from multi-target effects. Metal ions have been shown to play key roles in human development, enzyme activation, and metabolic homeostasis 16, 18. Therefore, the abnormal balance of metal ions can lead to many human diseases, including neurodegenerative diseases and cancer 16, 18. Studies have found that metallic iron, as a basic element of life necessary for bacteria, fungi, plants, and animals, binds with enzymes in the human body to promote hematopoiesis, blood coagulation, hormone maturation, and cell energy metabolism, and is involved in multiple biological behaviors 16, 18. However, excessive amounts of iron ions, especially divalent iron, will kill cells and cause pathological damage to multiple organs. Under normal circumstances, cells regulate their iron content through active homeostatic mechanisms to maintain a relatively constant level to prevent excessive iron accumulation and associated cell damage 16, 18. Cell damage and death associated with iron ions is known as ferroptosis 16, 18. Since the discovery of ferroptosis, iron ions have gradually gained attention as a research and development target for adjuvant drugs for tumor treatment, leading to the question of whether anisomycin also causes ferroptosis. This hypothesis has not yet been tested. We confirmed experimentally that the combination of anisomycin and the ferroptosis inhibitor ferrostatin-1 (Ferr-1) could significantly weaken the inhibitory effect of anisomycin on ovarian cancer stem cell proliferation. At the same time, the combination of anisomycin and the ferroptosis agonist Erastin killed more ovarian cancer stem cells than anisomycin alone. Anisomycin could also increase the contents of LPO, MDA, and Fe^2+^ in HuOCSCs, while decreasing the content of T-GSH. These substances are the necessary or key conditions for the occurrence of ferroptosis in cells. Therefore, we confirmed experimentally that anisomycin has the potential to induce ferroptosis.

Subsequently, we analyzed the changes in the expression levels of ferroptosis‑related signal transduction pathway genes in HuOCSCs before and after treatment with anisomycin, and the relationship between the expression levels of these genes in ovarian cancer tissues and tumor malignancy and prognosis. Studies have revealed a close relationship between autophagy and ferroptosis 19, 20, 36, 37. Autophagy is a process by which cells engulf their own cytoplasmic proteins or organelles and coat them into vesicles, where they fuse with lysosomes to form autophagolysosomes, which degrade their encapsulated contents, thereby fulfilling the metabolic needs of cells and the renewal of certain organelles 36, 38. Recent studies have reported that autophagy and ferroptosis are not two independent events; rather, they are inextricably linked 19, 20, 36-40. Autophagy and ferroptosis both promote and depend on each other, and the relationship between ferroptosis and autophagy is different in different individuals, cells, and diseases 19, 20, 36-40. An *et al.* loaded autophagy-promoting rapamycin onto MnO2@HMCu2-xS nanocomposites and transfected them into human breast cancer cells. They reported a positive correlation between ferroptosis and autophagy in these cells. The authors speculated that autophagy is an indispensable physiological phenomenon in the process of cellular ferroptosis 39. Wei *et al*. reported that arsenic could damage mitochondria and induce pancreatic dysfunction and ferroptosis by increasing mitochondrial ROS to induce an autophagy‑lysosomal disorder 37. However, Zhao also found that a 15‑lipoxygenase‑1-PE-binding protein-1-generated ferroptotic phospholipid, 15-proferroptotic hydroperoxy-arachidonoyl-phosphatidylethanolamine, promoted LC3-I lipidation to stimulate autophagy 38. This concurrent activation of autophagy protects cells from ferroptosis and the release of mitochondrial DNA. Therefore, under conditions of iron ion overload, the cellular autophagy level decreases, leading to the occurrence of ferroptotic death 38. In addition, Liu *et al.* provided evidence that the interplay between the signals of mechanistic target of rapamycin kinase (mTOR) and GPX4 modulates autophagy‑dependent ferroptosis in human pancreatic cancer cells, and the downregulated expression of GPX4 enhances the anti-cancer activity of rapamycin *in vitro* or *in vivo* by promoting ferroptosis and suppressing autophagy 38. In addition, our previous study demonstrated that superparamagnetic ferrite nanoparticles could significantly inhibit the autophagy activity of OCSCs, thereby promoting the occurrence of ferroptosis 19. Thus, autophagy and ferroptosis are closely related; however, the relationship between the two is not purely positive or negative, and different regulatory mechanisms occur according to different situations. In this study, PPI network analysis showed that the expression levels of members of the glutathione metabolism and autophagy signal transduction pathways were significantly lower in the process of anisomycin-induced HuOCSC ferroptosis. The experimental results suggested that the cytotoxicity of anisomycin is achieved by attenuating autophagy and glutathione metabolism. This also confirmed the intrinsic link between ferroptosis and autophagy, because glutathione metabolism is necessary to protect cells from ferroptosis.

Subsequently, we conducted in-depth data mining analysis of the relationship between the expression levels of core genes of the glutathione metabolism and autophagy signal transduction pathways and the malignancy degree and prognosis of ovarian cancer. The results demonstrated that the expression levels of multiple core genes of the above pathways were significantly different in patient-derived ovarian cancer tissues and normal tissues, and correlated significantly with poor prognosis of patients with ovarian cancer. This result provides a theoretical basis for future treatment, because if the induction of ferroptosis is used as an adjuvant drug for ovarian cancer treatment, the target protein must be significantly expressed to be stimulated by the corresponding drug. The glutathione metabolism and autophagy signal transduction pathways, as the core driving signaling pathways of ferroptosis, are crucial in the corresponding stimulation of iron ions and lipid peroxides. Our theoretical prediction indicated that the transcription and expression levels of core factors of the glutathione metabolism and autophagy signal transduction pathways are significantly different in tumor and healthy tissues. In addition, considering that the increased transcription level of a gene is closely related to the activation of transcription factors, we re-analyzed the cDNA microarray analysis data of anisomycin-treated HuOCSCs and confirmed that the expression level of *ATF4* was significantly reduced in the anisomycin-treated group. Bioinformatic analyses showed that *ATF4* expression correlated positively with the expression levels of *GPX4*, *GSS*, *KEAP1*, *NFE2L2*, *SLC7A11*, *ATG3*,* ATG4D*, and *ATG5*. Subsequent motif analysis confirmed that the promotors of *GPX4*, *GSS*, *NFE2L2*, *ATG3*, and *ATG4D* contain multiple ATF4 binding sites, and the basic sequences of the binding sites were consistent with the reported motif for ATF4. Therefore, we believe that the direct target of anisomycin-induced ferroptosis in HuOCSCs is *ATF4*. Anisomycin downregulated the expression and activity of ATF4, making it unable to activate the transcription and expression of the core factors of the glutathione metabolism and autophagy signal transduction pathways. Eventually, ovarian cancer stem cells lost their protection and underwent ferroptosis.

In addition, we performed exosome database screening and found that the levels of ATF4, GPX4, GSS, KEAP1, and ATG3 in the peripheral blood exosomes of patients with ovarian cancer were significantly higher than those from the healthy controls. Recently, studies have reported using the contents of exosomes in bodily fluids as non‑invasive diagnostic markers. Exosomes are formed by the release of intracellular endosomes into the extracellular space through exocytosis. Exosomes have diameters ranging from about 20 nm to 180 nm, and contain numerous cellular components, including DNA, RNA, lipids, metabolites, cytoplasmic proteins, and membrane surface proteins. Exosomes, as transport vehicles for intracellular substances, are abundant in peripheral blood and can be easily separated and enriched. Thus, they are a potential and sensitive source of non-invasive diagnostic markers. Our research indicated that by detecting the levels of ATF4, GPX4, GSS, KEAP1, and ATG3 in the peripheral blood of patients with ovarian cancer, their progression and prognosis could be rapidly, accurately, and effectively predicted, and their sensitivity to adjuvant therapy with ferroptosis-related drugs could be predicted.

## Conclusions

In summary, the results of the present study allowed us to hypothesize that anisomycin suppresses the expression of glutathione metabolism and autophagy signal transduction pathways by downregulating the expression of ATF4. In addition, anisomycin has the potential to induce human ovarian cancer stem cell ferroptosis. Meanwhile, this study, suggested that anisomycin has multiple targets and various mechanisms of action when inhibiting the activity of ovarian cancer stem cells.

## Supplementary Material

Supplementary figure S1.

## Abbreviations

ATF4: Activating transcription factor 4
JNK: JUN N-terminal kinase
Bcl2: B-cell CLL/lymphoma 2
CFLIP: CASP8 and FADD-like apoptosis regulator
Mcl1: Myeloid cell leukemia sequence 1
GPX4: Glutathione peroxidase 4
Fsp1: Ferroptosis suppressor protein 1
ROS: Reactive oxygen species
OCSCs: Ovarian cancer stem cells
Anisomycin: 3,4-Pyrrolidinediol,2-[(4-meth-oxyphenyl)methyl]-,3-acetate,(2R,3S,4S)
MTT: 3-(4,5-dimethylthiazol-2-yl)-2,5-diphenyltetrazolium-bromide
TBA: Thiobarbituric acid
ATP: Adenosine triphosphate
RLU: Relative light unit
GEPIA: Gene expression profiling interactive analysis
PANTHER: Protein Analysis through evolutionary relationships
GO: Gene Ontology
PPI: Protein-protein interaction network
qRT-PCR: quantitative real-time reverse transcription polymerase chain reaction
PBS: phosphate-buffered saline
