# Supplementary figures and images for: Anisomycin has the potential to induce human ovarian cancer stem cell ferroptosis by influencing glutathione metabolism and autophagy signal transduction pathways
Source: J Cancer. 2023 May 5;14(7):1202–15. doi: 10.7150/jca.83355 (PMC10197939; doi:10.7150/jca.83355)

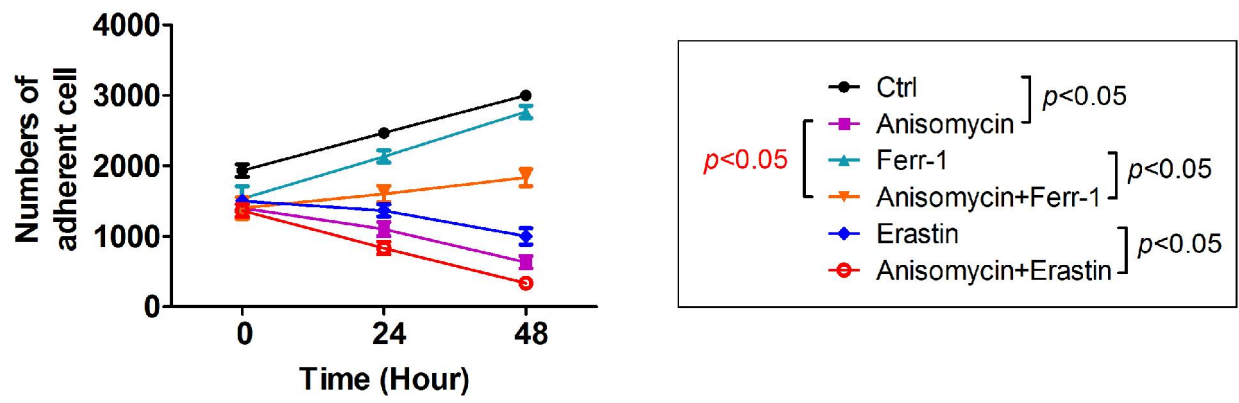

Figure S1. The counting result of adherent cells

Supplement: Supplementary file 1 — Supplementary figure S1. [file jcav14p1202s1.pdf]
